# Kikuchi-Fujimoto Disease Presenting as Necrotic Cervical Lymphadenopathy Mimicking Tuberculous Lymphadenitis in a Young Woman With Strong Familial Autoimmunity: A Case Report

**DOI:** 10.7759/cureus.119392

**Published:** 2026-11-09

**Authors:** Reshma David, Jinsha Khan Basheer

**Affiliations:** 1 Acute Medicine, Queen Elizabeth Hospital Birmingham, University Hospitals Birmingham NHS Foundation Trust, Birmingham, GBR

**Keywords:** autoimmunity, cervical lymphadenopathy, dsdna positivity, kikuchi-fujimoto disease, lymph node biopsy, necrotizing lymphadenitis, systemic lupus erythematosus, tuberculous lymphadenitis

## Abstract

Kikuchi-Fujimoto disease (KFD), also known as histiocytic necrotizing lymphadenitis, is a rare and usually self-limiting condition that predominantly affects young adults, particularly women. It commonly presents with fever and cervical lymphadenopathy and may be accompanied by weight loss, night sweats, and fatigue. Due to its nonspecific presentation, KFD can mimic infectious, malignant, and autoimmune conditions, making diagnosis challenging. We report the case of a 20-year-old woman with a significant family history of autoimmune disease who presented with a two-week history of intermittent fever, progressively painful left-sided cervical lymphadenopathy, weight loss, night sweats, and lethargy. Initial investigations revealed leukopenia and raised inflammatory markers, while computed tomography demonstrated bilateral cervical lymphadenopathy with a necrotic left cervical lymph node. Lymph node biopsy demonstrated necrotising lymphadenitis consistent with KFD. Subsequent investigations revealed positive anti-double-stranded DNA antibodies, negative antinuclear antibodies, and lupus-like clinical manifestations, raising the possibility of an evolving autoimmune disorder. This case highlights the importance of considering KFD in the differential diagnosis of young patients presenting with fever and cervical lymphadenopathy, particularly when autoimmune features and a significant family history are present. It also emphasises the importance of distinguishing KFD from lupus lymphadenitis and the need for ongoing rheumatological surveillance.

## Introduction

Kikuchi-Fujimoto disease (KFD), also known as histiocytic necrotizing lymphadenitis, is a rare and usually self-limiting condition that predominantly affects young adults, particularly women. The disease most commonly presents with fever and cervical lymphadenopathy and may be accompanied by constitutional symptoms, including weight loss, night sweats, and fatigue. Owing to its non-specific clinical presentation, KFD can mimic infectious, malignant, and autoimmune conditions, often resulting in extensive investigations before a definitive diagnosis is established [1-4].

The aetiology of KFD remains incompletely understood, although infectious and autoimmune mechanisms have been proposed. An association between KFD and systemic lupus erythematosus (SLE) has been increasingly recognised, with reports describing KFD preceding, occurring concurrently with, or developing following a diagnosis of SLE. Histopathological overlap between KFD and lupus lymphadenitis can further complicate the diagnostic process, making histological examination and clinicopathological correlation essential [3-8]. KFD generally follows a benign course, with spontaneous resolution occurring over several weeks to months. Management is primarily supportive, although corticosteroids or other immunomodulatory treatments may be considered in severe, persistent, or recurrent cases [9].

We report a case of KFD presenting with necrotic cervical lymphadenopathy mimicking tuberculous lymphadenitis, complicated by lupus-like manifestations and a significant family history of autoimmune disease. This case highlights the diagnostic challenges associated with KFD and emphasises the importance of considering underlying autoimmune conditions and ongoing rheumatological surveillance during follow-up.

## Case presentation

Initial presentation

A 20-year-old female university student presented with a two-week history of intermittent fever and progressively enlarging painful left-sided cervical swelling. Her symptoms began shortly after returning from international travel. She reported reduced appetite, lethargy, weight loss, and night sweats. There was no history of cough, haemoptysis, abdominal pain, urinary symptoms, rash, or recent illness contact. She also reported intermittent palpitations and episodes of dizziness associated with transient lower limb weakness and falls. She also reported previous contact with an individual diagnosed with tuberculosis.

Her past medical history included vitamin D deficiency and anxiety. Family history was notable for autoimmune conditions, including SLE and sarcoidosis, as well as multiple sclerosis and Kikuchi lymphadenitis.

On examination, she was febrile with a temperature of 39°C and tachycardic, with a heart rate of 142 beats/minute. An electrocardiogram (ECG) demonstrated sinus tachycardia. Chest radiography was normal, and high-sensitivity troponin I was less than 4. Lymph node examination revealed two to three palpable submental and superficial cervical lymph nodes, each small (estimated to be less than 1 cm in greatest diameter), discrete, mobile and tender to palpation. No axillary, supraclavicular or inguinal lymphadenopathy was identified. The remainder of the cardiovascular, respiratory, abdominal and neurological examinations was unremarkable.

Investigations

Initial laboratory investigations demonstrated leucopenia with a white cell count of 2.87 × 10⁹/L, neutropenia of 1.3 × 10⁹/L, and an elevated C-reactive protein (CRP) of 44 mg/L. Alanine aminotransferase was also elevated at 131 U/L. HIV, hepatitis B surface antigen, and hepatitis C antibody testing were negative. Serial haematological and biochemical results are summarised in Table 1.

**Table 1:** Serial laboratory investigations demonstrating the initial inflammatory response and subsequent haematological recovery. Bold values indicate abnormalities at initial presentation. Reference ranges are based on the reporting laboratory. CRP, C-reactive protein; eGFR, estimated glomerular filtration rate; ALT, alanine transaminase; ALP, alkaline phosphatase

Parameter	Day 0	Day 1	Day 2	Day 23	Day 45	Day 130
Haemoglobin (g/L)	129	125	—	120	127	126
White cell count (×10⁹/L)	2.87	3.36	—	3.18	8.25	3.90
Neutrophils (×10⁹/L)	1.30	1.50	—	1.40	6.11	1.70
Lymphocytes (×10⁹/L)	1.30	1.50	—	1.40	1.62	1.70
Platelets (×10⁹/L)	226	237	—	255	312	293
CRP (mg/L)	44	—	37	2	<1	<1
Sodium (mmol/L)	136	—	138	138	142	138
Potassium (mmol/L)	4.0	—	3.7	4.0	4.2	4.1
Creatinine (µmol/L)	54	—	44	49	74	52
eGFR (mL/min/1.73 m²)	>90	—	>90	>90	>90	>90
ALT (U/L)	131	—	125	118	87	79
ALP (U/L)	26	—	31	117	79	79
Albumin (g/L)	43	—	36	47	45	47

Given the constitutional symptoms, cervical lymphadenopathy, cytopenia, and concern for an underlying systemic inflammatory or autoimmune disorder, further investigations were performed to evaluate alternative diagnoses, including SLE, haematological disease, and infectious causes. The autoimmune and immunological investigations are summarised in Table 2.

**Table 2:** Autoimmune, immunological and infectious investigations during diagnostic evaluation. Autoimmune, immunological, and infectious investigations performed to evaluate alternative causes of cervical lymphadenopathy and cytopenia, including systemic lupus erythematosus, haematological disease, and infection, showed that anti-double-stranded DNA (anti-dsDNA) was elevated, while antinuclear antibodies (ANA) and extractable nuclear antigen (ENA) screening were negative and complement levels were within the laboratory reference ranges. Serum protein electrophoresis demonstrated a polyclonal pattern without evidence of a monoclonal protein. Tuberculosis PCR, acid-fast bacilli staining, and mycobacterial culture of the lymph-node specimen were negative. CMV IgG and EBV nuclear antigen IgG positivity were consistent with previous exposure. Reference ranges are based on the reporting laboratory. anti-CCP, anti-cyclic citrullinated peptide; CMV, cytomegalovirus; EBV, Epstein–Barr virus; ENA, extractable nuclear antigen; HIV, human immunodeficiency virus; PCR, polymerase chain reaction

Investigation	Result	Interpretation
Antinuclear antibodies (ANA)	Not detected	Negative
Anti-double-stranded DNA (anti-dsDNA)	15.0 IU/mL	Above reference range
Extractable nuclear antigen (ENA) screen	Negative	Negative
Complement C3	1.09 g/L	Within reference range
Complement C4	0.28 g/L	Within reference range
Immunoglobulin G (IgG)	17.44 g/L	Above reference range
Immunoglobulin A (IgA)	0.73 g/L	Within reference range
Immunoglobulin M (IgM)	1.75 g/L	Within reference range
Serum protein electrophoresis	Polyclonal	No monoclonal pattern identified
Monoclonal protein	Not detected	Not detected
Immunofixation	No abnormality detected	No monoclonal protein identified
Anti-CCP antibodies	Negative	Negative
HIV Ag/Ab	Negative	Negative
Hepatitis B surface antigen	Negative	Negative
Hepatitis C antibody	Negative	Negative
Tuberculosis PCR, lymph node	*M. tuberculosis* complex not detected	Negative
Mycobacterial culture, lymph node	Negative	No mycobacterial growth
Acid-fast bacilli stain, lymph node	Not seen	Negative
Toxoplasma IgM	Negative	No evidence of recent infection
Toxoplasma IgG	Negative	No evidence of previous exposure
CMV IgM	Negative	No evidence of recent infection
CMV IgG	Positive	Previous exposure
EBV nuclear antigen (EBNA) IgG	Positive	Previous exposure

CT of the neck, thorax, abdomen, and pelvis demonstrated pathological bilateral cervical lymphadenopathy involving levels II-IV and left level V, including a necrotic left level II lymph node. Splenomegaly measuring 16 cm was also identified. No significant mediastinal, hilar, abdominal, or pelvic lymphadenopathy was present. Although the abdominal examination was unremarkable, splenomegaly was demonstrated on CT imaging. The radiological appearance was considered highly concerning for tuberculous lymphadenitis (Figure 1).

**Figure 1:**
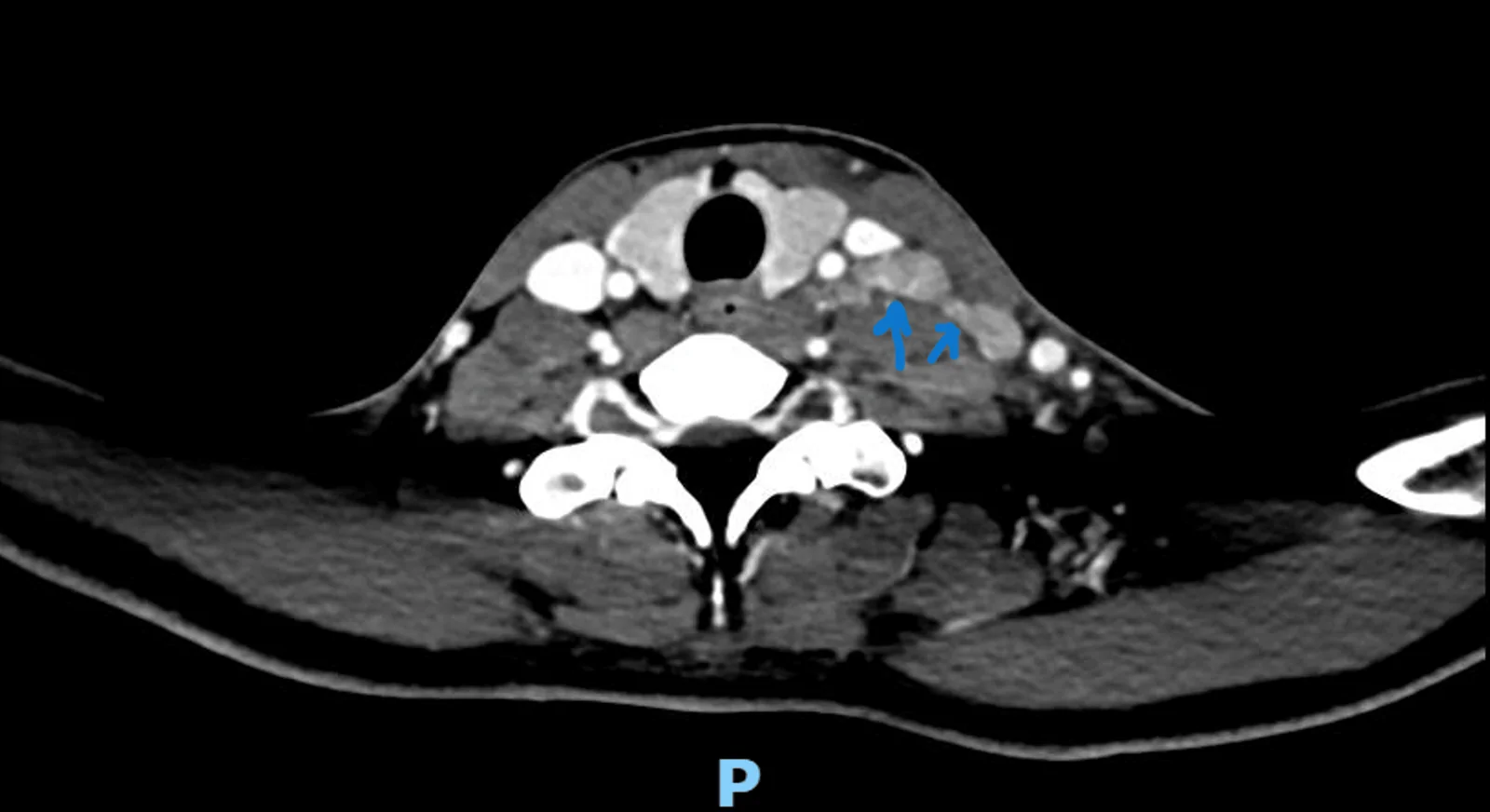
Contrast-enhanced CT demonstrating bilateral cervical lymphadenopathy involving levels II–IV and left level V, with central necrosis of a left level II lymph node (arrow).

Given the constitutional symptoms, previous tuberculosis exposure, necrotic lymphadenopathy, and splenomegaly, extensive investigations for tuberculosis were undertaken. Acid-fast bacilli (AFB) were not identified on lymph node microscopy. *Mycobacterium tuberculosis* complex DNA was not detected on GeneXpert (Cepheid, Sunnyvale, California, United States) polymerase chain reaction (PCR) testing of the lymph node specimen. Mycobacterial culture of the lymph node and tuberculosis blood cultures were subsequently negative.

Histopathological findings

An ultrasound-guided core needle biopsy was selected as a less invasive approach to obtain tissue for histopathological and immunohistochemical assessment. Although excisional biopsy generally provides more complete lymph-node architecture and may be preferred when lymphoma or other causes of lymphadenopathy are suspected, a core biopsy was performed in this case. Histological examination demonstrated fragmented lymph node tissue with patchy foci of early necrosis containing karyorrhectic debris, histiocytoid cells, and abundant CD8-positive T lymphocytes (Figure 2).

**Figure 2:**
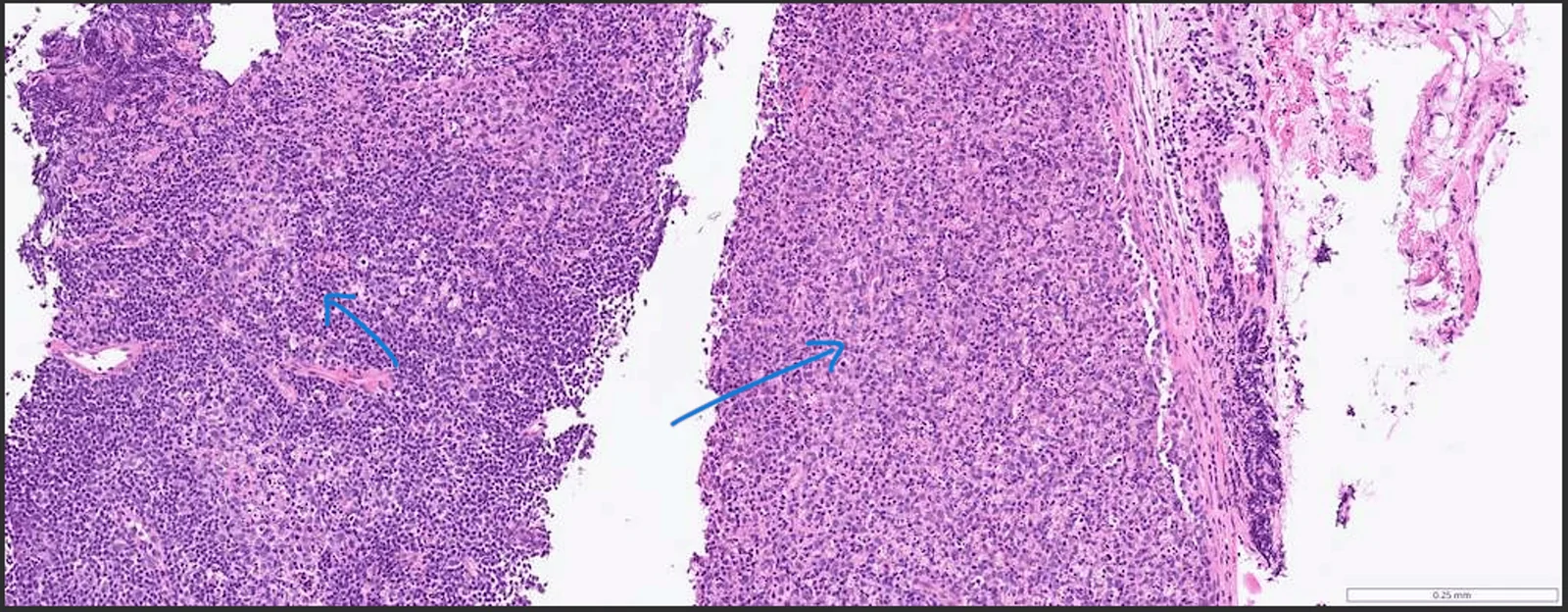
Histopathological features of necrotising lymphadenitis in a cervical lymph-node biopsy. Haematoxylin and eosin (H&E)-stained section of a left cervical lymph node demonstrating patchy necrosis with karyorrhectic debris and an associated histiocytic and lymphocytic infiltrate, consistent with necrotising lymphadenitis.

No mature neutrophils, granulomas, organisms, or Hodgkin/Reed-Sternberg-like cells were identified.

Immunohistochemistry demonstrated MPO-positive and lysozyme-positive histiocytes and monocytes admixed with CD123-positive plasmacytoid dendritic cells. B-cell and T-cell populations were preserved without evidence of lymphoma. Special stains, including periodic acid-Schiff (PAS), diastase-periodic acid-Schiff (DPAS), and AFB stains, were negative for microorganisms. The overall findings were considered suggestive of Kikuchi lymphadenitis, although lupus lymphadenitis could not be completely excluded.

Re-presentation and rheumatology assessment

Three weeks following discharge, the patient re-presented with persistent constitutional symptoms and new polyarthralgia involving the shoulders, elbows, and wrists, associated with morning stiffness. She also reported exertional dyspnoea and intermittent pleuritic chest pain.

Further review revealed a previous history of photosensitive facial rash that had been evaluated several years earlier. Clinical examination demonstrated persistent cervical lymphadenopathy but no objective synovitis, active cutaneous rash, or other documented clinical features suggestive of connective tissue disease.

Repeat investigations showed significant improvement in inflammatory markers, with CRP falling to 2 mg/L and subsequently less than 1 mg/L. Autoimmune testing revealed persistent anti-double-stranded DNA (anti-dsDNA) antibody positivity (15.0 IU/mL initially and 15.0 IU/mL on repeat testing), negative antinuclear antibody testing, negative extractable nuclear antigen screening, and normal complement levels (C3 1.09 g/L and C4 0.28 g/L). Urinalysis was normal.

Diagnosis, management, and follow-up

The patient was reviewed by the rheumatology team. In view of the biopsy findings, negative tuberculosis investigations, absence of significant organ involvement, and lack of clinical evidence supporting an alternative diagnosis, KFD was considered the most likely diagnosis.

She was commenced on prednisolone 15 mg daily for two weeks for symptomatic relief and remains under rheumatology follow-up, currently receiving a tapering dose of prednisolone. Given the positive anti-dsDNA antibody, previous history of photosensitive rash, and strong family history of autoimmune disease, ongoing surveillance for the possible future development of SLE was recommended.

## Discussion

KFD and diagnostic challenges

KFD is a rare, benign and usually self-limiting form of necrotising lymphadenitis that predominantly affects young adults, particularly women. It commonly presents with cervical lymphadenopathy, fever, and constitutional symptoms, including weight loss and night sweats. These non-specific clinical features can closely resemble infectious, malignant and autoimmune conditions, making diagnosis challenging [1-3,9].

Although the pathogenesis of KFD remains incompletely understood, an abnormal immune-mediated response involving cytotoxic T lymphocytes and histiocytes is thought to play an important role. Infectious triggers and autoimmune mechanisms have both been proposed [2,7-9]. Histologically, KFD is characterised by patchy necrosis, karyorrhectic debris and an infiltrate rich in histiocytes and CD8-positive T lymphocytes, typically with an absence of neutrophils. Differentiation from lupus lymphadenitis can be difficult because of overlapping histopathological features, highlighting the importance of correlating biopsy findings with clinical and serological investigations [2,4,5,9].

Our patient presented with fever, painful cervical lymphadenopathy, weight loss, night sweats, leucopenia, and splenomegaly. CT demonstrated bilateral pathological cervical lymphadenopathy, including a necrotic lymph node, raising significant concern for tuberculous lymphadenitis. Given the clinical presentation and history of tuberculosis exposure, tuberculosis was initially considered a leading diagnosis. This case illustrates how KFD can closely mimic tuberculous lymphadenitis and reinforces the need to investigate potentially serious infectious and malignant causes before establishing a diagnosis of KFD.

Role of histopathology in establishing the diagnosis

Histopathological examination is central to diagnosing KFD, particularly when clinical and radiological findings are suggestive of alternative conditions. Characteristic features include patchy areas of necrosis containing karyorrhectic debris, histiocytic infiltration and plasmacytoid dendritic cells, with an absence or marked paucity of neutrophils [2,6-9].

In our patient, cervical lymph node biopsy demonstrated patchy necrosis with karyorrhectic debris and MPO-positive and lysozyme-positive histiocytes, supporting a diagnosis of KFD. Plasmacytoid dendritic cells were also identified, while no granulomas or microorganisms were seen. Extensive microbiological investigations, including acid-fast bacilli staining, mycobacterial culture, and tuberculosis PCR, were negative. Together, the histopathological findings and negative microbiological investigations supported KFD over tuberculous lymphadenitis.

This case highlights the importance of obtaining tissue for histopathological assessment in patients with persistent cervical lymphadenopathy and constitutional symptoms. Clinical and radiological findings alone may be insufficient to distinguish KFD from tuberculosis, lymphoma or other causes of necrotising lymphadenitis, potentially leading to inappropriate treatment or delayed diagnosis [2,6,9].

Association between KFD and SLE

The association between KFD and SLE is well recognised, although the relationship remains incompletely understood. KFD may precede, occur concurrently with, or develop after the diagnosis of SLE. Furthermore, the two conditions can share clinical and histopathological features, making differentiation challenging in some patients [4,5,9].

Several features in our patient raised concern for an underlying or evolving connective tissue disease. These included a history of photosensitive facial rash, repeated anti-dsDNA positivity, constitutional symptoms, polyarthralgia, and a maternal history of SLE. However, ANA and extractable nuclear antigen testing remained negative, complement levels were normal, and there was no evidence of renal or other major organ involvement. Following rheumatological assessment, the overall clinical and histopathological findings were considered most consistent with KFD rather than established SLE. The isolated anti-dsDNA positivity should therefore be interpreted in the context of the negative ANA, other serological findings, and absence of convincing clinical evidence of SLE.

The family history was also notable, with SLE in the patient's mother, sarcoidosis in her father, multiple sclerosis in a maternal aunt and KFD in a cousin. Although this clustering of immune-mediated conditions is striking, a direct genetic relationship cannot be established from a single case. It may, however, suggest a possible familial susceptibility to immune dysregulation and provides an additional reason for careful clinical follow-up.

Despite the absence of a definitive diagnosis of SLE, ongoing rheumatological surveillance is appropriate given the recognised association between KFD and autoimmune disease. Long-term follow-up is important because SLE or other autoimmune manifestations may become clinically apparent months or years after the initial presentation [1,4,5,9].

Clinical implications and management

KFD is generally self-limiting, with many patients recovering spontaneously over several weeks to months. Management is therefore usually supportive, with anti-inflammatory treatment for symptom control. Corticosteroids may be considered in patients with severe, persistent, or recurrent symptoms [1,2,9]. In our patient, a short course of prednisolone was prescribed for ongoing symptomatic disease, alongside rheumatological follow-up.

This case demonstrates the diagnostic challenges posed by KFD when it presents with necrotic cervical lymphadenopathy and constitutional symptoms suggestive of tuberculosis. It highlights the importance of excluding serious alternative diagnoses, particularly infection and malignancy, and the diagnostic value of lymph node histopathology. It also emphasises the need to assess for associated autoimmune disease and maintain appropriate follow-up when clinical or serological features raise concern for evolving SLE.

## Conclusions

KFD should be considered in young patients presenting with fever, cervical lymphadenopathy, and constitutional symptoms, even when radiological findings raise concern for tuberculosis. As KFD can mimic serious infectious and malignant conditions, careful exclusion of alternative diagnoses and histopathological assessment are essential. This case also highlights the importance of recognising possible autoimmune associations and arranging appropriate long-term rheumatological follow-up, particularly in patients with lupus-like features or a significant family history of autoimmunity.
